# Performance of C-reactive protein and procalcitonin to distinguish viral from bacterial and malarial causes of fever in Southeast Asia

**DOI:** 10.1186/s12879-015-1272-6

**Published:** 2015-11-11

**Authors:** Yoel Lubell, Stuart D. Blacksell, Susanna Dunachie, Ampai Tanganuchitcharnchai, Thomas Althaus, Wanitda Watthanaworawit, Daniel H. Paris, Mayfong Mayxay, Thomas J. Peto, Arjen M. Dondorp, Nicholas J. White, Nicholas P.J. Day, François Nosten, Paul N. Newton, Paul Turner

**Affiliations:** Mahidol-Oxford Tropical Medicine Research Unit (MORU), Faculty of Tropical Medicine, Mahidol University, Bangkok, Thailand; Centre for Tropical Medicine and Global Health, Nuffield Department of Medicine, University of Oxford, Oxford, UK; Shoklo Malaria Research Unit (SMRU), Mahidol-Oxford Tropical Medicine Research Unit, Faculty of Tropical Medicine, Mahidol University, Mae Sot, Thailand; Lao-Oxford-Mahosot Hospital-Wellcome Trust Research Unit (LOMWRU), Mahosot Hospital, Vientiane, Laos; Faculty of Postgraduate Studies, University of Health Sciences, Vientiane, Laos; Cambodia Oxford Medical Research Unit, Angkor Hospital for Children, Siem Reap, Cambodia

**Keywords:** Bacterial infections, Fever, Sepsis, Procalcitonin, C reactive protein, Malaria, Sensitivity and specificity, Southeast Asia

## Abstract

**Background:**

Poor targeting of antimicrobial drugs contributes to the millions of deaths each year from malaria, pneumonia, and other tropical infectious diseases. While malaria rapid diagnostic tests have improved use of antimalarial drugs, there are no similar tests to guide the use of antibiotics in undifferentiated fevers. In this study we estimate the diagnostic accuracy of two well established biomarkers of bacterial infection, procalcitonin and C-reactive protein (CRP) in discriminating between common viral and bacterial infections in malaria endemic settings of Southeast Asia.

**Methods:**

Serum procalcitonin and CRP levels were measured in stored serum samples from febrile patients enrolled in three prospective studies conducted in Cambodia, Laos and, Thailand. Of the 1372 patients with a microbiologically confirmed diagnosis, 1105 had a single viral, bacterial or malarial infection. Procalcitonin and CRP levels were compared amongst these aetiological groups and their sensitivity and specificity in distinguishing bacterial infections and bacteraemias from viral infections were estimated using standard thresholds.

**Results:**

Serum concentrations of both biomarkers were significantly higher in bacterial infections and malaria than in viral infections. The AUROC for CRP in discriminating between bacterial and viral infections was 0.83 (0.81–0.86) compared with 0.74 (0.71–0.77) for procalcitonin (*p* < 0.0001). This relative advantage was evident in all sites and when stratifying patients by age and admission status. For CRP at a threshold of 10 mg/L, the sensitivity of detecting bacterial infections was 95 % with a specificity of 49 %. At a threshold of 20 mg/L sensitivity was 86 % with a specificity of 67 %. For procalcitonin at a low threshold of 0.1 ng/mL the sensitivity was 90 % with a specificity of 39 %. At a higher threshold of 0.5 ng/ul sensitivity was 60 % with a specificity of 76 %.

**Conclusion:**

In samples from febrile patients with mono-infections from rural settings in Southeast Asia, CRP was a highly sensitive and moderately specific biomarker for discriminating between viral and bacterial infections. Use of a CRP rapid test in peripheral health settings could potentially be a simple and affordable measure to better identify patients in need of antibacterial treatment and part of a global strategy to combat the emergence of antibiotic resistance.

## Background

Millions of deaths in the tropics each year follow a bacterial, viral, or malarial infection that often initially present as undifferentiated fever [1]. Many febrile patients seek care at peripheral levels of the healthcare system where diagnostic capacities are minimal. Given that malaria and bacterial infections may be rapidly fatal, overtreatment with antimicrobials is common, contributing to drug resistance that threatens global public health [2–4].

Widespread deployment of malaria rapid tests revolutionized the management of fever, until then treated presumptively with antimalarials [5]. It also revealed the role of many non-malarial infections as common causes of illness, highlighting the challenges in their diagnosis and treatment. In many endemic areas where malaria transmission is usually low and seasonal such as Southeast Asia, the majority of fevers will have a negative malaria test. While health workers can refrain safely from providing antimalarials to these patients, they have limited capacity to diagnose and treat other causes of illness. This compromises immediate health outcomes as well as patients’ confidence and future utilisation of public health services.

There are numerous challenges to diagnosis and treatment of infectious diseases in these settings. First, the limited epidemiological evidence available indicates high spatial and temporal heterogeneity in incidence of infections such as leptospirosis, dengue and rickettsioses [6]. Second, community and low level health facility workers often have minimal training and have only recently emerged from a mind-set that all fevers should be treated presumptively with antimalarials [7]. Third, and perhaps most important, there are few point of care tests, other than malaria rapid diagnostic tests (RDTs), which are sufficiently accurate and appropriate for these settings. Syndromic based treatment algorithms such as the Integrated Management of Childhood Illness (IMCI) provide simple guidelines to improve the targeting of antibiotics and referral decisions; however evaluations of IMCI have shown mixed results in achieving this [8].

An alternative approach is to use biomarkers of inflammation to identify patients likely to benefit from antibiotics or referral to higher level facilities. Procalcitonin and C-reactive protein (CRP) are established biomarkers of bacterial infections, and are widely used in high income countries, particularly in hospital settings [9, 10]. In some countries point-of-care CRP tests are also routinely used in the community in patients with respiratory symptoms and have reduced the use of antibiotics safely and cost-effectively [11–13]. Extending this strategy to the tropics could reduce unnecessary use of antibiotics and curtail the spread of drug resistance. In low income countries this is particularly important as second line antimicrobials can be inaccessible. It remains critical, however, that patients with infections who could benefit from an antibiotic are identified correctly.

Thus far, there have been few comparative assessments of procalcitonin and CRP in malaria endemic areas, and those available have focused on hospitalised patients [14–16]. In this study we carry out a preliminary exploration of the role these biomarkers might have in peripheral settings in Southeast Asia to inform the use of antibiotics in non-malarial fevers by estimating their diagnostic accuracy in samples with well-defined viral and bacterial infections.

## Methods

Based on evidence from other settings [10, 17–22], we hypothesised that procalcitonin and CRP serum levels would be significantly higher in bacterial infections than in viral infections in malaria endemic areas of Southeast Asia. We tested this hypothesis using samples from patients with acute undifferentiated fever among whom an infection was identified as the likely cause of illness.

### Samples used in the study

We used stored serum samples from three aetiology of fever studies carried out in the Angkor Hospital for Children in Cambodia [23], in two provincial hospitals in rural Laos [24], and in rural clinics on the Thai-Myanmar border (manuscript in preparation; details summarised in Table 1). In all three studies the samples used for both microbiological investigations and for subsequent procalcitonin and CRP measurement were obtained on initial presentation, with convalescence samples used to confirm diagnoses. Procalcitonin and CRP were not routinely used for diagnosis and treatment in these sites or to inform study recruitment. In the Cambodian study a microbiological cause was identified in almost 50 % of paediatric inpatients, in Laos 41 % of a mixed inpatient and outpatient population (5 to 49 years of age) were assigned a ‘conservative’ diagnosis (using only culture, antigen detection, PCR, and for JEV IgM ELISA), as were 51 % of clinic attendees in the Thai-Myanmar border (children ≥5 years and adults of all ages). Test strategies for each of the studies are summarised in the supplementary file. Cases with a single microbiological diagnosis were assigned to one of four general aetiological groups: − viral infections, rickettsia, and leptospira infections, bacteraemias, and malaria. Our main interest was in the ability of the biomarkers to distinguish between infections in the viral aetiological group from those in either of the two bacterial aetiological groups, as well as from the bacteraemia group in isolation. We used 100 samples from healthy blood donors from a blood bank in Bangkok, Thailand as controls to characterise procalcitonin and CRP levels in a healthy population in the region.

**Table 1:** Study sites and participants from whom stored samples were obtained

	Cambodia	Laos	Thai/Myanmar border
Recruitment sites	Paediatric hospital	Two provincial hospitals in northwest and southern Laos	Migrant and refugee clinics on the Thai/Myanmar border
Enrolment dates	October 2009 to October 2010	May 2008 to December 2010; September 2008 to December 2010	March 2011 to March 2013
Patients recruited	*n* = 1180	*n* = 1938	*n* = 1029
Patient demographics	<16 years (69 % < 5); 45 % female; All inpatients	5–49 years (37 % < 15); 42 % female; 44 % inpatients	≥5 years (45 % < 15 years); 36 % female; clinic attendants
Symptoms/syndromes used for inclusion/exclusion	Documented axillary temperature ≥38 °C within 48 h of admission. All febrile inpatients were eligible irrespective of symptoms/syndromes but excluding post-surgical cases	Fever (tympanic ≥38 °C) with no obvious cause < 8 days and eligible for a malaria test by Laos national guidelines (patients with obvious causes of fever such as abscess or severe diarrhoea were excluded)	Documented fever ≥38 °C of up to seven days duration with no obvious cause. Patients with a clear clinical diagnosis such as chickenpox, pneumonia (based on clinical criteria), skin/soft tissue infection (e.g. cellulitis), or urinary tract infection were excluded
Documented mortality	5.6 %	0.5 %	0.1 %
Organisms tested for	*Plasmodium* spp, *Leptospira* spp, *O tsutsugamushi*, *R typhi*, spotted-fever-group rickettsia, agents of conventional bacteraemia, dengue fever, Japanese encephalitis virus, influenza	*Plasmodium* spp, *Leptospira* spp, *O tsutsugamushi*, *R typhi*, spotted-fever-group rickettsia, causes of community bacteraemia, dengue fever, Japanese encephalitis virus, influenza (in one of the two sites)	*Plasmodium* spp, *Leptospira* spp, *O tsutsugamushi*, *R typhi*, spotted-fever-group rickettsia, causes of community bacteraemia, dengue fever, Japanese encephalitis virus

### Laboratory procedures

The VIDAS® B · R · A · H · M · S PCT kit was used to determine procalcitonin levels using the Enzyme-Linked Fluorescent Assay technique via the Mini-VIDAS platform (BioMérieux, 69280 Marcy-l'Etoile, France). The assay has a lower level of detection of 0.05 ng/ml and a measurement range of 0.05–195 ng/ml. CRP was measured using a NycoCard Reader II (Axis Shield, Kjelsåsveien 161, NO-0884 Oslo, Norway). The assay has an effective range of 5–120 mg/L in serum and has been validated for accuracy in both laboratory and routine care settings [25, 26]. All serum samples from the original studies were stored at −85 °C before testing. While steps were taken to prevent multiple freeze-thaw cycles, we used five samples from the Cambodia study to further confirm the stability of procalcitonin and CRP levels over repeated freeze-thaw cycles. In approximately 20 % of cases there were insufficient volumes of serum for the procalcitonin assay therefore these cases were excluded. All procalcitonin and CRP assay results were read independently by two operators blinded to the microbiological diagnosis, and double-entered to minimise omissions or inaccuracies.

### Statistical analysis

Procalcitonin and CRP values were summarised using medians and ranges. These were compared across aetiological groups using the Mann-Whitney *U* test for two-group comparisons and the Kruskal-Wallis test for multi-group comparisons. Non-parametric receiver operating characteristic (ROC) curves were plotted and the Wald test was used to compare areas under the curve. Covariates included the following factors: patient admission (inpatients or outpatients), prior use of antibiotics, and age group. The analyses were conducted for each of the studies in isolation, and by merging data from all studies and controlling for site effect in estimating the area under the receiver operating curve (AUROC) values.

To classify normal or elevated procalcitonin levels we used established thresholds of 0.1 ng/mL, recommended for lower acuity patients, as defined by the facility level they attend and their admission status, and a more widely used threshold of 0.5 ng/mL [21, 27]. For CRP we used a threshold of 10 mg/L and 20 mg/L [17, 28]. Sensitivity, specificity and likelihood ratios of procalcitonin and CRP in detecting bacterial infections from a mixed pool with viral infections were calculated with 95 % confidence intervals using the Wilson Score method. The analysis was performed using STATA 13 (College Station, Texas).

### Ethical approval

Ethical approval for the original studies was obtained from the Angkor Hospital for Children Institutional Review Board (IRB) in Cambodia, the Lao National Ethics Committee for Health Research and the Mahidol University Faculty of Tropical Medicine Ethics Committee in Thailand, and from the Oxford Tropical Research Ethics Committee (OXTREC). In all three sites patients or their guardian signed an informed consent prior to inclusion in the fever study; in the Laos and Thailand studies this included use of the samples for future investigations with similar aims. In Cambodia we obtained further approval from the hospital IRB as the original patient consent form did not specify that other investigations with similar aims could be performed. OXTREC was consulted and no further ethical review was required.

## Results

**Fig. 1:**
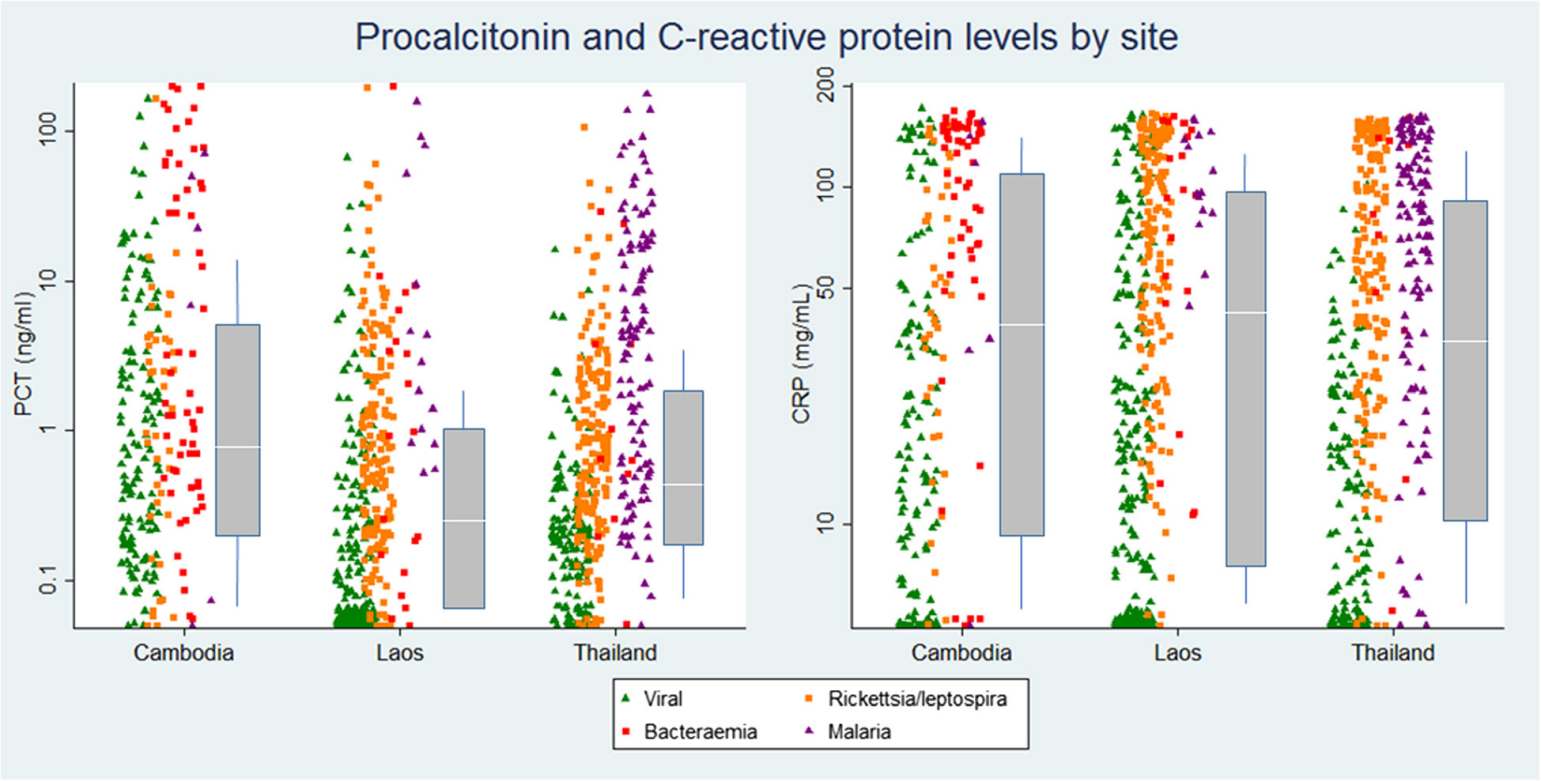
Boxplots for the distribution of procalcitonin and CRP readings in the three sites, and data points for procalcitonin and CRP levels in viral infections, rickettsia/leptospira, bacteraemia and malaria. Abbreviation: PCT, Procalcitonin; CRP, C reactive protein

In total, 1372 patient samples were available for analysis. Procalcitonin levels were significantly higher in the Cambodian hospital, with a study population of paediatric inpatients and mortality exceeding 5 %, than in the other two community based sites where there was a broader age distribution, a majority of outpatients and mortality under 0.5 % (*p* < 0.001) (Fig. 1). For CRP there were no significant differences between the sites. Among the 100 controls, none had a raised procalcitonin level and two had moderately raised CRP levels (10 and 31 mg/L).

Of the 1372 samples, 267 had multiple infections identified. Samples with a single diagnosis (*n* = 1105) were classified into the four general aetiological groups: − viral infections, rickettsia, and leptospira infections, bacteraemias, and malaria (Tables 2 and 3, Fig. 1). There was no significant difference in gender ratio in each group. In the Cambodian study, children under 5 years were more likely to have a viral aetiology than older children (68 % against 52 %, *p* < 0.001).

**Table 2:** Samples excluded from the analysis due to insufficient volume and the samples included in the analysis by site

Cause of fever	Excluded due to low volume n (%)	Cambodia n (%)	Laos n (%)	Thailand n (%)	Total n (%)	Aetiological group (% of samples with single infection)
Dengue	98 (25)	81 (19)	75 (14)	157 (37)	313 (23)	Viral, *n* = 555 (50)
Japanese encephalitis	38 (10)	26 (6)	81 (16)	5 (1)	112 (8)	
Influenza/RSV	23 (6)	32 (8)	98 (19)	NA	130 (9)	
Rickettsial infection^a^	56 (15)	36 (8)	89 (17)	92 (21)	217 (16)	Rickettsia/Leptospira, *n* = 334 (30)
Leptospirosis	39 (10)	7 (2)	70 (14)	40 (9)	117 (9)	
Bacteraemia	46 (12)	59 (14)	20 (4)	11 (3)	91 (7)	Bacteraemia, *n* = 91 (8)
Malaria	10 (3)	6 (1)	15 (3)	104 (24)	125 (9)	Malaria, *n* = 125 (12)
Multiple pathogens	73 (19)	179 (42)	68 (13)	21 (5)	267 (19)	
Total	383	426 (100)	516 (100)	433 (100)	1372 (100)	1105

These samples were a sub-set of the original study, to include only those with identified infections. The main focus of the analysis was on those samples with a single pathogen that was identified as the cause of illness (allowing for co-infection with malaria PCR positive microscopy/RDT negative cases which are not assumed to be the cause of fever)

^a^Scrub or murine typhus

**Table 3:** Samples with pathogenic organisms isolated in blood culture

Samples with pathogenic organisms isolated in blood cultures	Cambodia n (%)	Laos n (%)	Thailand n (%)	TotaL n (%)
*Acinetobacter bacteraemia*	1 (2)			1 (1)
*B. pseudomallei*	6 (10)			6 (7)
*E. coli*	4 (7)	2 (10)	5 (46)	11 (12)
*H. influenzae*	4 (7)			4 (4)
*K. pneumoniae*	1 (2)			1 (1)
*P. aeruginosa*	1 (2)	1 (5)		2 (2)
*S. aureus*	18 (31)			18 (20)
*S. pneumoniae*	12 (20)		1 (9)	13 (15)
*S. pyogenes*	2 (3)			2 (2)
*S.* Typhi */ S.* Paratyphi	9 (15)	17 (85)	3 (27)	28 (32)
*Salmonella spp.*	1 (2)		1 (9)	2 (2)
*Enterococcus sp.*			1 (9)	2 (2)

Only samples for which sufficient volumes were available and without evidence of co-infections were included in the analysis

**Fig. 2:**
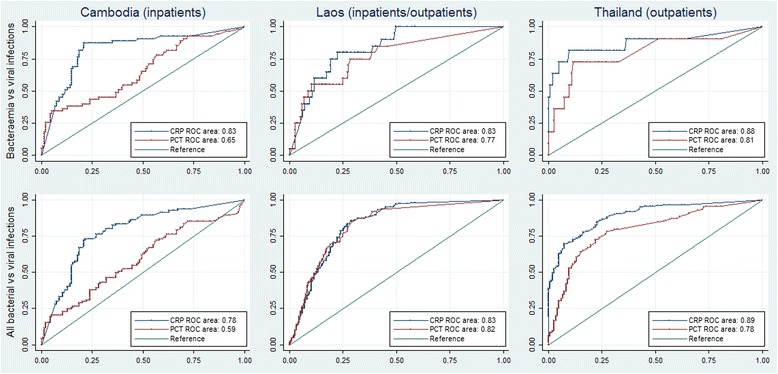
Receiver operating characteristic curves for procalcitonin and CRP in discriminating between aetiological groups. On the horizontal axis is the sensitivity of the biomarkers and on the vertical axis is the false positive rate (1-specificity). Abbreviations: PCT, Procalcitonin; CRP, C reactive protein; ROC, receiver operating characteristic curve

Procalcitonin and CRP levels were significantly lower in patients with a viral infection than other infections (*p* < 0.0001 in all comparisons apart from procalcitonin values in the Cambodian site where this was *p* = 0.01 for the difference between viral and bacterial infections). The AUROC for CRP [0.83 (0.81–0.86)] in discriminating between all bacterial and viral infections was significantly higher than that for procalcitonin [0.74 (0.71–0.77)] (*p* < 0.0001). When including only bacteraemias and viral infections CRP had an AUROC of 0.89 (0.8–0.97) as compared with 0.78 for procalcitonin (0.65–0.90) in distinguishing between the two aetiological groups (Fig. 2).

**Fig. 3:**
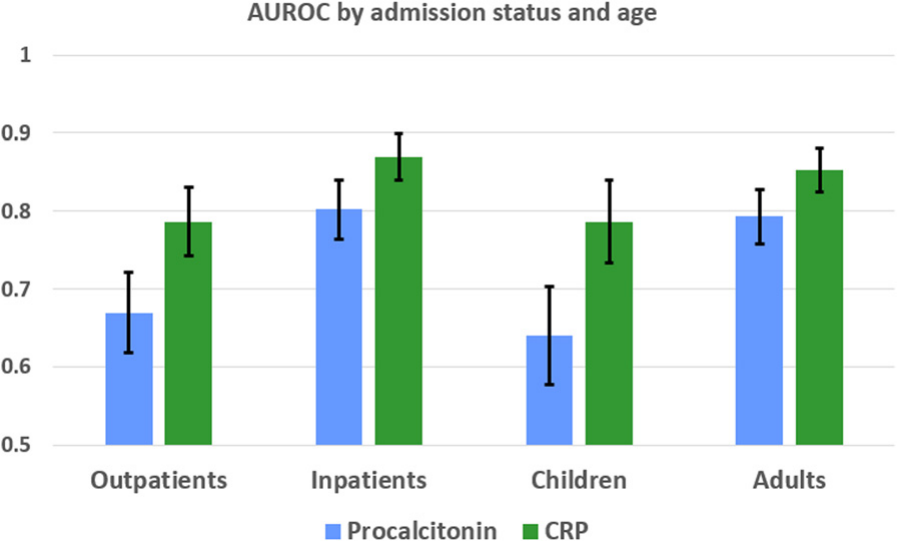
AUROC for Procalcitonin and C-reactive protein levels by admission status and age group

The samples included in this study were obtained from a diverse group of patients. When controlling for covariates (age group, admission status, previous use of antibiotics), the AUROC for CRP remained significantly higher than that of procalcitonin. Being admitted as an inpatient had an independent effect of elevated levels of procalcitonin (*p* = 0.054) and CRP (*p* = 0.006). In a stratified analysis by patient age group and by admission status the AUROC for CRP remained statistically higher than that for procalcitonin, as shown in Fig. 3.

**Fig. 4:**
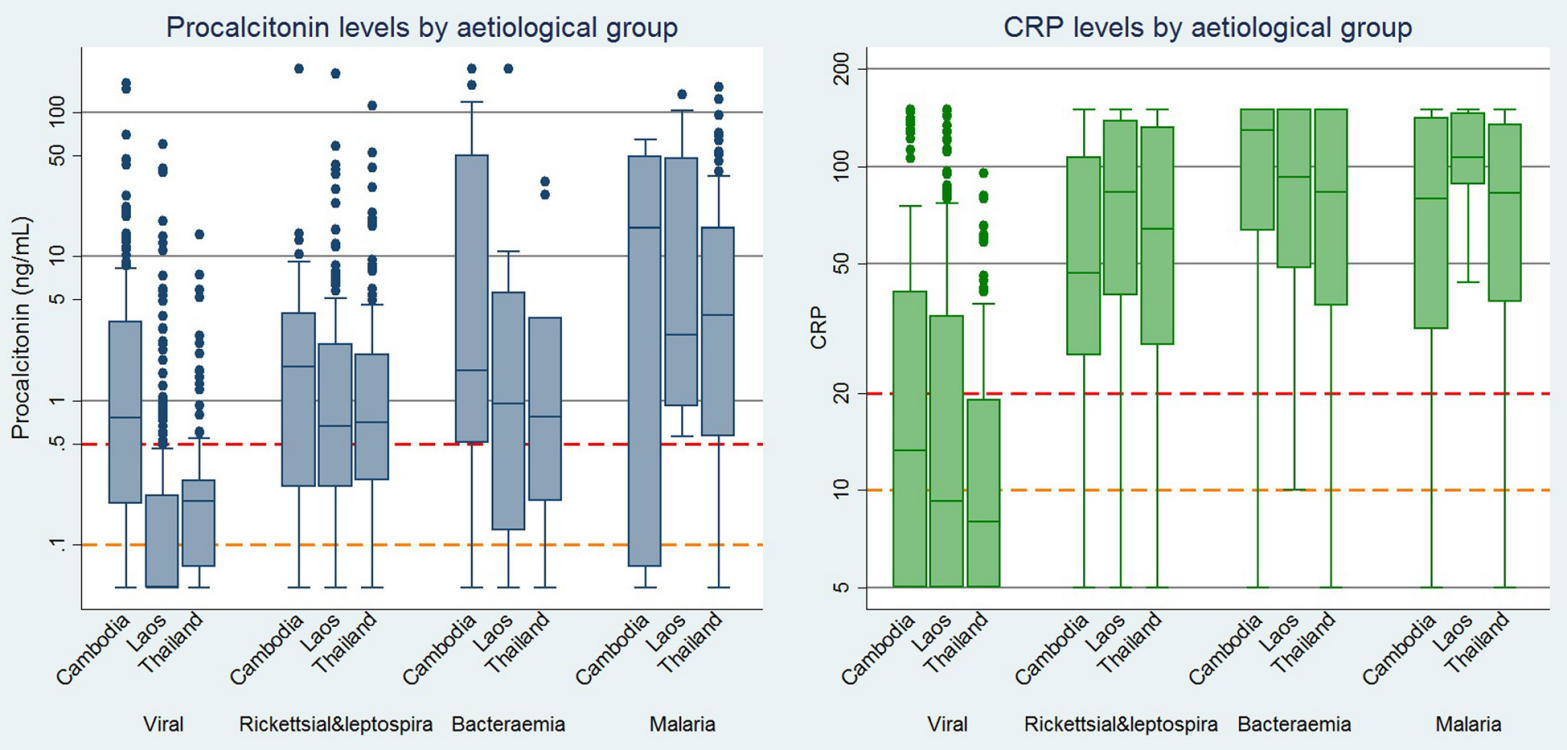
Procalcitonin and C-reactive protein levels in the four aetiological groups by site. Abbreviation: AUROC—area under the receiver operating curve; CRP—C-reactive protein

The distribution of procalcitonin and CRP levels in each of the aetiological groups with respect to the threshold values are shown in Fig. 4. When compared against their respective threshold values of 0.1 ng/mL or 0.5 ng/mL (procalcitonin) and 10 mg/L or 20 mg/L (CRP), elevated procalcitonin predicted the presence of bacterial infection with a sensitivity of 90 % (87–92 %) and a specificity of 39 % (35–43 %) using the lower threshold of 0.1 ng/mL, or a sensitivity of 60 % (56–65 %) and specificity of 76 % (72–79 %) for the higher threshold of 0.5 ng/ml. For CRP at a threshold of 10 mg/L the sensitivity was 95 % (92–97 %) and the specificity was 49 % (46–53 %); at a 20 mg/L threshold the sensitivity was 86 % (82–88 %) and specificity was 67 % (63–71 %). The moderate positive likelihood ratio of 1.9 for the lower CRP threshold therefore implies limited additional benefit in confirming the presence of bacterial infections, but the negative likelihood ratio of 0.1 implies high confidence in ruling out the need for antibiotics. The proportion of samples with elevated procalcitonin and CRP levels by aetiology are shown in Table 4.

**Table 4:** Percentage of patients with procalcitonin and C-reactive protein above threshold values

Aetiology	PCT 0.1 ng/ml	PCT 0.5 ng/ml	CRP 10 mg/L	CRP 20 mg/L
	%, 95 % CI	%, 95 % CI	%, 95 % CI	%, 95 % CI
Dengue	72 (67–77)	25 (21–31)	46 (40–52)	28 (23–33)
Japanese encephalitis	60 (50–69)	32 (24–41)	64 (55–73)	52 (43–62)
Influenza	34 (26–42)	16 (11–24)	49 (40–57)	29 (22–37)
Rickettsial infections	87 (82–91)	53 (47–60)	94 (90–97)	80 (75–85)
Leptospirosis	95 (91–98)	65 (57–72)	97 (92–99)	92 (87–96)
Bacteraemia	90 (82–95)	71 (60–80)	93 (85–97)	86 (77–92)
Malaria	96 (91–98)	79 (72–85)	93 (88–97)	87 (80–92)

In viral infections the proportion of patients with a raised procalcitonin level was consistently and significantly lower than that for CRP at 10 mg/L (*p* < 0.001). For the higher CRP threshold of 20 mg/L the differences in specificity were not significant, except in the case of Japanese encephalitis

*Abbreviation*: *PCT* Procalcitonin; *CRP* C reactive protein

Procalcitonin and CRP levels were significantly higher in malaria infections as compared with viral infections (*p* < 0.001). For CRP levels there was no significant difference between bacterial infections and malaria (*p* = 0.15) while procalcitonin levels were significantly higher (*p* < 0.001). The AUROC value for procalcitonin in discriminating between malaria and bacterial infections was 0.69 (95 % CI 0.64–0.74) and for CRP it was 0.54 (0.49–0.6).

In all sites, median procalcitonin and CRP levels for samples with more than one identified pathogen were higher than in those with a single pathogen, but these differences were non-significant. In Cambodia, where procalcitonin and CRP assays were carried out on samples without an identified pathogen there was no evidence of a difference compared with samples with an identified pathogen.

## Discussion

This is the largest ever study to measure and compare procalcitonin and C-reactive protein levels in malaria endemic settings. While both biomarkers were elevated in malaria and bacterial infections as compared with viral infections, CRP was the more accurate biomarker for predicting the presence of bacterial infections in these samples. In addition, CRP holds pragmatic advantages as it rises to higher concentrations in the presence of infection, which may allow more accurate and low cost point of care tests that rely on low volumes of whole blood [29]. The commercial availability of CRP rapid tests [30, 31] and their routine use in high-income countries in community and hospital settings suggests that the timeframe for possible adoption elsewhere would be considerably shorter. Of these two well-established biomarkers we therefore consider CRP to be more promising for further evaluation and development for the context of primary care in the rural tropics.

Malaria is associated with elevated CRP [28, 32] and the data from this study as well as the limited previous evidence suggest this is also the case for procalcitonin [14]. These biomarkers therefore have limited value in guiding antibiotic treatment in parasitaemic individuals, at least in the Southeast Asian setting. In an African high transmission setting, a positive malaria RDT may not be a reliable indication of malaria being the cause of illness, particularly with histidine-protein D 2 antigen based RDTs in severe cases [33, 34]; possible use of a CRP test in malaria RDT positives in these settings warrants further investigation.

In low transmission settings most infections are symptomatic and detected by malaria RDTs, but if a significant proportion of the population is asymptomatically infected with sub-patent infections and if this is associated with elevated CRP/ procalcitonin, this could compromise the utility of these biomarkers in guiding the management of fevers in these settings. Further work is currently being carried out to measure CRP levels in asymptomatic, slide negative infections detected by highly sensitive PCR.

In the literature comparing procalcitonin and CRP as biomarkers of bacterial infection, procalcitonin was often found superior, particularly in critical care [10, 35, 36]. This could be due to a faster reaction time in response to infection, which could be advantageous in initiating treatment as quickly as possible. Procalcitonin is also more rapidly eliminated, which can be beneficial in guiding antibiotic termination in hospitalised patients. These advantages however might be less apparent in the context of community care in the rural tropics where access to treatment is inherently slower and where health workers contact with patients is less frequent. Of more relevance to this context is the literature concerning primary care and emergency department (ED) settings. These have shown more variable results in terms of the comparative ability of the two biomarkers to identify bacterial infections [28, 37, 38]. One of the few available comparisons of PCT and CRP in non-malarial tropical diseases was from the very different population of ill-returned travellers, where CRP was also found to be superior to procalcitonin in patients in discriminating between a confirmed viral infection (dengue) and those with either typhoid or rickettsioses [39].

Ultimately the utility of these and other biomarkers needs to be determined not in retrospective studies on samples with imperfect diagnoses but in prospective studies into their impact on clinical outcomes and antibiotic prescription. Meta-analyses of procalcitonin guided treatment in respiratory infections (as opposed to febrile patients in our study) found it to safely reduce the use of antibiotics with no significant impact on mortality, ICU admission or length of hospital stay [40, 41]. A recent Cochrane review of biomarkers to guide antibiotics in ARIs in primary care concluded that CRP was the only sufficiently accurate biomarker for which point of care tests are available that could safely and effectively reduce the prescribing of antibiotics [17]. Most studies concluded that the utility of these biomarkers was greatest when interpreted in conjunction with clinical symptoms and other laboratory assessments. It is important to emphasise that the context of patient care in the rural tropics is very different—health workers often have minimal training or experience, and no access to other laboratory tests.

There are several limitations that need to be addressed in further study. First, we included stored samples with a single conservative diagnosis from a subset of patients with undifferentiated fevers. Likewise, the performance of the biomarkers could differ if other viral and bacterial infections that were not investigated in the original studies were included in the groupings. The implications for patients without a confirmed microbiological diagnosis or with other pathogens are not clear. These findings therefore are likely to be of high internal validity but their generalizability needs to be better established in a prospective cohort of consecutively recruited patients. Ultimately establishing a microbiological cause of illness will always be challenging, therefore in addition to comparing biomarker levels in patients for whom a diagnosis is available, what is most needed are studies exploring antibiotic prescription patterns and clinical outcomes when guided by CRP or procalcitonin in these settings [42].

Second, there was variation between the studies in the microbial pathogens investigated, laboratory procedures, and algorithms used to determine a cause of illness. The inclusion criteria also differed in terms of whether all febrile patients were included or only those without a clear focus of infection. However, the consistent performance of CRP across the different studies indicates that the findings are robust.

Third, the accuracy of the microbiological diagnoses where available was likely to be imperfect. It is possible that patients with an identified viral infection and elevated procalcitonin or CRP might also be harbouring an undetected pathogenic bacterial infection, as documented for example in hypotensive dengue patients with translocation of gut bacteria [43]. While this detracts from the apparent accuracy of the tests in misclassifying bacterial infections as viral, in a routine setting the implication would be that these cases are appropriately treated with an antibiotic.

Fourth, there was considerable diversity in patient profile including age and severity of illness between the sites; the Cambodian site for instance included only paediatric inpatients as compared with the Thai/Myanmar study, which included primarily older outpatients. Our main objective is to inform the management of fever in community and primary care, therefore some of these patient profiles might be more reflective of severe cases attending higher level facilities. In practice however, such distinctions might not hold in remote areas where severely ill patients often present and remain at low level facilities.

Lastly, the thresholds used to define elevated procalcitonin and CRP levels might not be optimal; specifying an optimal threshold requires further clinical and economic evaluation.

## Conclusions

Notwithstanding the need for further prospective studies of CRP guided treatment in these settings, these findings suggest that there may well be a role for CRP point of care tests in guiding antibiotic treatment of fevers in malaria endemic areas. A key advantage of this approach is its simplicity. Malaria RDTs are already widely used and therefore incorporation of a test with a similar format, preferably in a single device will not require extensive further infrastructure or training. As compared with using a range of aetiology specific tests, a single biomarker test is also likely to have lower costs and greater robustness to temporal and spatial heterogeneity in fever aetiology. Other biomarkers and technologies are under development and some of these will undoubtedly eventually prove superior to a CRP immunoassay. Current management of undifferentiated fever in the rural tropics however is unacceptable, with dire health outcomes from common treatable infections going undiagnosed, while overconsumption of antibiotics contributes to a potential global public health catastrophe. We believe the addition of CRP rapid tests to current syndromic based treatment algorithms could be a feasible, effective, and affordable measure to improve healthcare delivery across the tropics; further work to confirm this approach is warranted.

## Abbreviations

CRP: C-reactive protein
IMCI: Management of Childhood Illness
PCT: Procalcitonin
RDT: Rapid diagnostic tests
